# Activation, Impaired Tumor Necrosis Factor-α Production, and Deficiency of Circulating Mucosal-Associated Invariant T Cells in Patients with Scrub Typhus

**DOI:** 10.1371/journal.pntd.0004832

**Published:** 2016-07-27

**Authors:** Seung-Ji Kang, Hye-Mi Jin, Eun Jeong Won, Young-Nan Cho, Hyun-Ju Jung, Yong-Soo Kwon, Hae Jin Kee, Jae Kyun Ju, Jung-Chul Kim, Uh Jin Kim, Hee-Chang Jang, Sook-In Jung, Seung-Jung Kee, Yong-Wook Park

**Affiliations:** 1 Department of Infectious Diseases, Chonnam National University Medical School and Hospital, Gwangju, Republic of Korea; 2 Department of Rheumatology, Chonnam National University Medical School and Hospital, Gwangju, Republic of Korea; 3 Department of Laboratory Medicine, Chonnam National University Medical School and Hospital, Gwangju, Republic of Korea; 4 Department of Pulmonary and Critical Care Medicine, Chonnam National University Medical School and Hospital, Gwangju, Republic of Korea; 5 Heart Research Center, Chonnam National University Hospital, Gwangju, Republic of Korea; 6 Department of Surgery, Chonnam National University Medical School and Hospital, Gwangju, Republic of Korea; University of Liverpool, UNITED KINGDOM

## Abstract

**Background:**

Mucosal-associated invariant T (MAIT) cells contribute to protection against certain microorganism infections. However, little is known about the role of MAIT cells in *Orientia tsutsugamushi* infection. Hence, the aims of this study were to examine the level and function of MAIT cells in patients with scrub typhus and to evaluate the clinical relevance of MAIT cell levels.

**Methodology/Principal Findings:**

Thirty-eight patients with scrub typhus and 53 health control subjects were enrolled in the study. The patients were further divided into subgroups according to disease severity. MAIT cell level and function in the peripheral blood were measured by flow cytometry. Circulating MAIT cell levels were found to be significantly reduced in scrub typhus patients. MAIT cell deficiency reflects a variety of clinical conditions. In particular, MAT cell levels reflect disease severity. MAIT cells in scrub typhus patients displayed impaired tumor necrosis factor (TNF)-α production, which was restored during the remission phase. In addition, the impaired production of TNF-α by MAIT cells was associated with elevated CD69 expression.

**Conclusions:**

This study shows that circulating MAIT cells are activated, numerically deficient, and functionally impaired in TNF-α production in patients with scrub typhus. These abnormalities possibly contribute to immune system dysregulation in scrub typhus infection.

## Author Summary

Scrub typhus is a mite-borne bacterial infection in humans caused by *Orientia tsutsugamushi*, an obligate intracellular bacterium, prevalent in Asia, Northern Australia, and the Indian subcontinent. The pathogenesis of *O*. *tsutsugamushi* infection is known to be not only related to the virulence of *O*. *tsutsugamushi*, but also to the host immune response. Mucosal-associated invariant T (MAIT) cells are an evolutionarily conserved antimicrobial MR1-restricted T cell subset. Upon antigen recognition, MAIT cells rapidly produce proinflammatory cytokines, maintain an activated phenotype throughout the course of an infection, and have the potential to directly kill infected cells; thus, playing an important role in controlling the host response. However, little is known about the role of MAIT cells in *Orientia tsutsugamushi* infection. To the best of our knowledge, this is the first study to measure the levels and functions of circulating MAIT cells in scrub typhus patients and to examine the clinical relevance of MAIT cell levels. The present study demonstrates that circulating MAIT cells are activated and numerically deficient in patients with scrub typhus. Notably, impairment of TNF-α production represents the susceptibility of individuals to *O*. *tsutsugamushi* infection. These findings provide important information for predicting the prognosis of scrub typhus infection.

## Introduction

Scrub typhus is a mite-borne bacterial infection in humans caused by *Orientia tsutsugamushi*, an obligate intracellular bacterium, prevalent in Asia, Northern Australia, and the Indian subcontinent. With early diagnosis and management, most patients with scrub typhus are able to recover without complications [1]. However, some patients develop serious and potentially fatal complications such as interstitial pneumonia, acute renal failure, myocarditis, meningoencephalitis, gastrointestinal bleeding, acute hearing loss, and multiple organ failure [2–4]. As the primary targets of *O*. *tsutsugamushi* are endothelial cells, the variable extent of vasculitis in each individuals helps in part to explain the different levels of severity [5]. However, a previous study has shown that diffuse alveolar damage could present without evidence of vasculitis, suggesting that the immunologic response plays a significant role in development of the disease and determination of the severity of illness [3].

The immune response induced by *O*. *tsutsugamushi* is a combination of innate and adaptive immunity and the proper response of macrophages and T lymphocytes may be the driving factor in immunity in patients with scrub typhus [5]. Furthermore, several studies reported dysfunction of the immunologic response of the host to *O*. *tsutsugamushi*; dysregulated levels of certain cytokines, imbalance of Th1/Th2 cytokines and apoptosis of T lymphocytes during acute infection [6–9]. These findings suggested that the pathogenesis of *O*. *tsutsugamushi* infection is not only related to the virulence of *O*. *tsutsugamushi*, but also to the host immune response.

Mucosal-associated invariant T (MAIT) cells are a relatively newly recognized T cell subset that expresses a conserved invariant T cell receptor (TCR) α-chain (Vα7.2-Jα33 in humans and Vα19-Jα33 in mice) paired with a limited set of Vβ chains [10]. Human MAIT cells are defined as CD3^+^TCRδγ^-^Vα7.2^+^CD161^high^ or CD3^+^TCRδγ^-^Vα7.2^+^IL-18Rα^+^ cells [11,12]. MAIT cells recognize bacteria-derived riboflavin (vitamin B2) metabolites presented by the MHC class 1b-like related protein (MR1) [10,13]. Upon antigen recognition, MAIT cells rapidly produce proinflammatory cytokines, such as interferon (IFN)-γ, tumor necrosis factor (TNF)-α, and interleukin (IL)-17, in an innate-like manner [14]. MAIT cells maintain an activated phenotype throughout the course of an infection, secrete inflammatory cytokines, and have the potential to directly kill infected cells; thus, playing an important role in controlling the host response [11,12,15–19]. However, little is known about the role of MAIT cells in *O*. *tsutsugamushi* infection. Accordingly, the aims of this study were to examine the level and function of MAIT cells in patients with scrub typhus and to evaluate the clinical relevance of MAIT cell levels.

## Methods

### Study subjects

The study cohort comprised 38 patients with scrub typhus (25 women and 13 men; mean age ± SD, 64.3 ± 15.6 years) and 53 healthy controls (HCs; 30 women and 23 men; mean age ± SD, 63.6 ± 12.2 years). All patients were confirmed as having scrub typhus by the serologic test using a passive hemagglutination assay (PHA) to detect *O*. *tsutsugamushi* antigen. A positive result was defined as a titer of ≥ 1:80 in a single serum sample or at least a fourfold rise in antibody titer at follow-up examination. PHA was performed using Genedia Tsutsu PHA II test kits (GreenCross SangA, Yongin, Korea). Scrub typhus patients were further divided into subgroups according to disease severity as previously described [7]. Patients with no organ dysfunction were considered to have mild disease, those with one organ dysfunction were considered to have moderate disease, while those with dysfunction of two or more organ systems were defined as having severe disease. Organ dysfunction was defined as follows: (1) renal dysfunction, creatinine ≥ 2.5 mg/dL; (2) hepatic dysfunction, total bilirubin ≥ 2.5 mg/dL; (3) pulmonary dysfunction, bilateral pulmonary infiltration on chest X-rays with moderate to severe hypoxia (PaO2/FiO2 < 300 mmHg or PaO2 < 60 mmHg or SpO2 < 90%); (4) cardiovascular dysfunction, systolic blood pressure < 80 mmHg despite fluid resuscitation; and (5) central nervous system dysfunction, significantly altered sensorium with Glasgow Coma Scale (GCS) ≤ 8/15. None of the controls had a history of autoimmune disease, infectious disease, malignancy, chronic liver or renal disease, diabetes mellitus, immunosuppressive therapy, or fever within 72 hours prior to enrollment.

### Ethics statement

The study protocol was approved by the Institutional Review Board of Chonnam National University Hospital, and written informed consent was obtained from all participants in accordance with the Declaration of Helsinki.

### Monoclonal antibodies (mAbs) and flow cytometry

The following mAbs and reagents were used in this study: Allophycocyanin (APC)-Cy7-conjugated anti-CD3, phycoerythrin (PE)-Cy5-conjugated anti-CD161 and fluorescein isothiocyanate (FITC)-conjugated anti-TCR γδ, FITC-conjugated anti-CD3, FITC-conjugated anti-IFN-γ, FITC-conjugated annexin V, PE-conjugated anti-CD3, PE-conjugated anti-IL-17, PE-Cy7-conjugated anti-TNF-α, PE-conjugated anti-CD69, FITC-conjugated mouse IgG isotype, PE-conjugated mouse IgG isotype and PE-Cy7-conjugated mouse IgG isotype control (all from Becton Dickinson, San Diego, CA); PE-conjugated anti-programmed death-1 (anti-PD-1; eBioscience, San Diego, CA) and APC-conjugated anti-TCR Vα7.2 (BioLegend, San Diego, CA). Cells were stained with combinations of appropriate mAbs for 20 minutes at 4°C. Stained cells were analyzed on a Navios flow cytometer using Kaluza software (version 1.1; Beckman Coulter, Brea, CA).

### Isolation of peripheral blood mononuclear cells (PBMCs) and identification of MAIT cells

Peripheral venous blood samples were collected in heparin-containing tubes, and PBMCs were isolated by density-gradient centrifugation using Lymphoprep (Axis-Shield PoC AS, Oslo, Norway). MAIT cells were identified phenotypically as CD3+TCRγδ-Vα7.2+CD161^high^ cells, by flow cytometry as previously described [20,21]. Total lymphocyte numbers were measured by Coulter LH750 automatic hematology analyzer (Beckman Coulter, Miami, FL). Absolute numbers of MAIT cells were calculated by multiplying the MAIT cell percentages by the CD3+γδ- T cell percentages and the total lymphocyte numbers (per microliter) in peripheral blood.

### Functional MAIT cell assay

IFN-γ, IL-17, and TNF-α expression in MAIT cells was detected by intracellular cytokine flow cytometry as previously described [11,12,21]. Briefly, freshly isolated PBMCs (1 × 10^6^/well) were incubated in 1 mL complete media, consisting of RPMI 1640, 2 mM _L_-glutamine, 100 units/mL of penicillin, and 100 μg/mL of streptomycin, and supplemented with 10% fetal bovine serum (FBS; Welgene, Gyeongsan, Korea) for 4 hours in the presence of phorbol myristate acetate (PMA) (100 ng/mL; Sigma, St Louis, MO) and ionomycin (IM) (1 μM; Sigma). For intracellular cytokine staining, 10 μL of brefeldin A (GolgiPlug; BD Biosciences, San Diego, CA) was added, and the final concentration of brefeldin A was 10 μg/mL. After incubation for additional 4 hours, cells were stained with APC-Cy7-conjugated anti-CD3, PE-Cy5-conjugated anti-CD161, and APC-conjugated anti-TCR Vα7.2 mAbs for 20 minutes at 4°C, fixed in 4% paraformaldehyde for 15 minutes at room temperature, and permeabilized using Perm/Wash solution (BD Biosciences) for 10 minutes. Cells were then stained with FITC-conjugated anti-IFN-γ, PE-conjugated anti-IL-17 and PE-Cy7-conjugated anti-TNF-α mAbs for 30 minutes at 4°C and analyzed by flow cytometry.

To determine changes in expression levels of CD69, annexin V and PD-1 in MAIT cells after stimulation with IL-12 and IL-18, freshly isolated PBMCs (1 × 10^6^/well) were incubated in 1 mL complete media for 24 hours in the presence of IL-12 (50 ng/mL; Miltenyi biotec, Bergisch Gladbach, Germany) and IL-18 (50 ng/mL; Medical and Biological Laboratories, Woburn, MA). Cells were then stained with FITC-conjugated anti-CD3, FITC-conjugated annexin V, APC-conjugated anti-TCR Vα7.2, PE-conjugated anti-CD3, PE-conjugated anti-CD69, PE-conjugated anti-PD-1 and PE-Cy5-conjugated anti-CD161 mAbs for 20 minutes at 4°C. CD69+, annexin V+, and PD-1+ MAIT cell levels were determined by flow cytometry.

### Statistical analysis

All comparisons of percentages and absolute numbers of MAIT cells, their cytokine levels, and expression levels of CD69, PD-1 and annexin V were analyzed using the Mann-Whitney U tests or paired t test. Linear regression analysis was used to test associations between MAIT cell levels and clinical or laboratory parameters. The Wilcoxon matched-pairs signed rank test was used to compare changes in MAIT cell levels and functions according to disease activity. *P* values less than 0.05 were considered statistically significant. Statistical analyses and graphic works were performed using SPSS version 18.0 software (SPSS, Chicago, IL) and GraphPad Prism version 5.03 software (GraphPad Software, San Diego, CA), respectively.

## Results

### Subject characteristics

The clinical and laboratory characteristics of scrub typhus patients are summarized in Table 1. Thirty-eight patients were included in this study. Of the 38 scrub typhus patients, 26 patients (68.4%) had mild disease; 9 patients (23.7%) had moderate disease; and 3 patients (7.9%) had severe disease. Eighteen patients were monitored longitudinally from the active state (before antibiotic therapy) to the remitted state (defined as resolution of all presenting symptoms after antibiotic therapy).

**Table 1:** Clinical and laboratory characteristics of 38 scrub typhus patients.

Variables	Scrub typhus	Healthy control
Age (years), mean ± SD	64.3 ± 15.6	63.6 ± 12.2
male/female, n	13/25	23/30
Clinical variables, n (%)		
Fever	38 (100)	0 (0)
Rash	21 (55.3)	0 (0)
Eschar	33 (86.8)	0 (0)
Confusion	3 (7.9)	0 (0)
Severity of disease, n (%)		
Mild disease	26 (68.4)	0 (0)
Moderate to severe disease	12 (31.6)	0 (0)
Renal dysfunction	3 (7.9)	0 (0)
Hepatic dysfunction	0 (0)	0 (0)
CNS dysfunction	0 (0)	0 (0)
Respiratory dysfunction	9 (23.7)	0 (0)
Circulatory dysfunction	3 (7.9)	0 (0)
Co-morbid conditions, n (%)		
Diabetes mellitus	9 (23.7)	0 (0)
Cardiovascular disease	3 (7.9)	0 (0)
Chronic kidney disease	1 (2.6)	0 (0)
Chronic hepatic disease	5 (13.2)	0 (0)
Malignancy	2 (5.3)	0 (0)
Laboratory variables, mean ± SD		
Leukocyte count (cells/μL)	8408 ± 4806	6278 ± 1720
Lymphocyte count (cells/μL)	1686 ± 1285	2157 ± 568.9
Hemoglobin level (g/dL)	12.0 ± 2.1	13.5 ± 1.3
Neutrophil count (cells/μL)	5812 ± 4100	3495 ± 1377
Platelet count (×10^3^ cells/μL)	146 ± 66.2	223 ± 46.5
Total bilirubin level (mg/dL)	0.7 ± 0.3	0.7 ± 0.23
Total protein level (g/dL)	6.1 ± 0.6	7.4 ± 0.6
Albumin level (g/dL)	3.2 ± 0.6	4.5 ± 0.4
AST level (U/L)	109 ± 81.3	20.9 ± 4.8
ALT level (U/L)	103 ± 80.7	13.3 ± 5.0
Alkaline phosphatase level (U/L)	128 ± 79.6	52.6 ± 14.4
LDH level (U/L)	808 ± 241	375 ± 58
CRP level (mg/dL)	8.33 ± 7.08	0.07 ± 0.14
ESR level (mm/hour)	27.9 ± 26.3	9.29 ± 4.46
Time at hospital visit (days)*, mean ± SD	5.2 ± 0.5	

*Abbreviations*: ALT: alanine aminotransferase; AST: aspartate aminotransferase; CNS, central nervous system; CRP: C-reactive protein; ESR: erythrocyte sedimentation rate; LDH: lactate dehydrogenase; n: number; SD: standard deviation.

* indicates time from symptom onset to antibiotic therapy.

### Reduced numbers of circulating MAIT cells in scrub typhus patients

**Fig 1:**
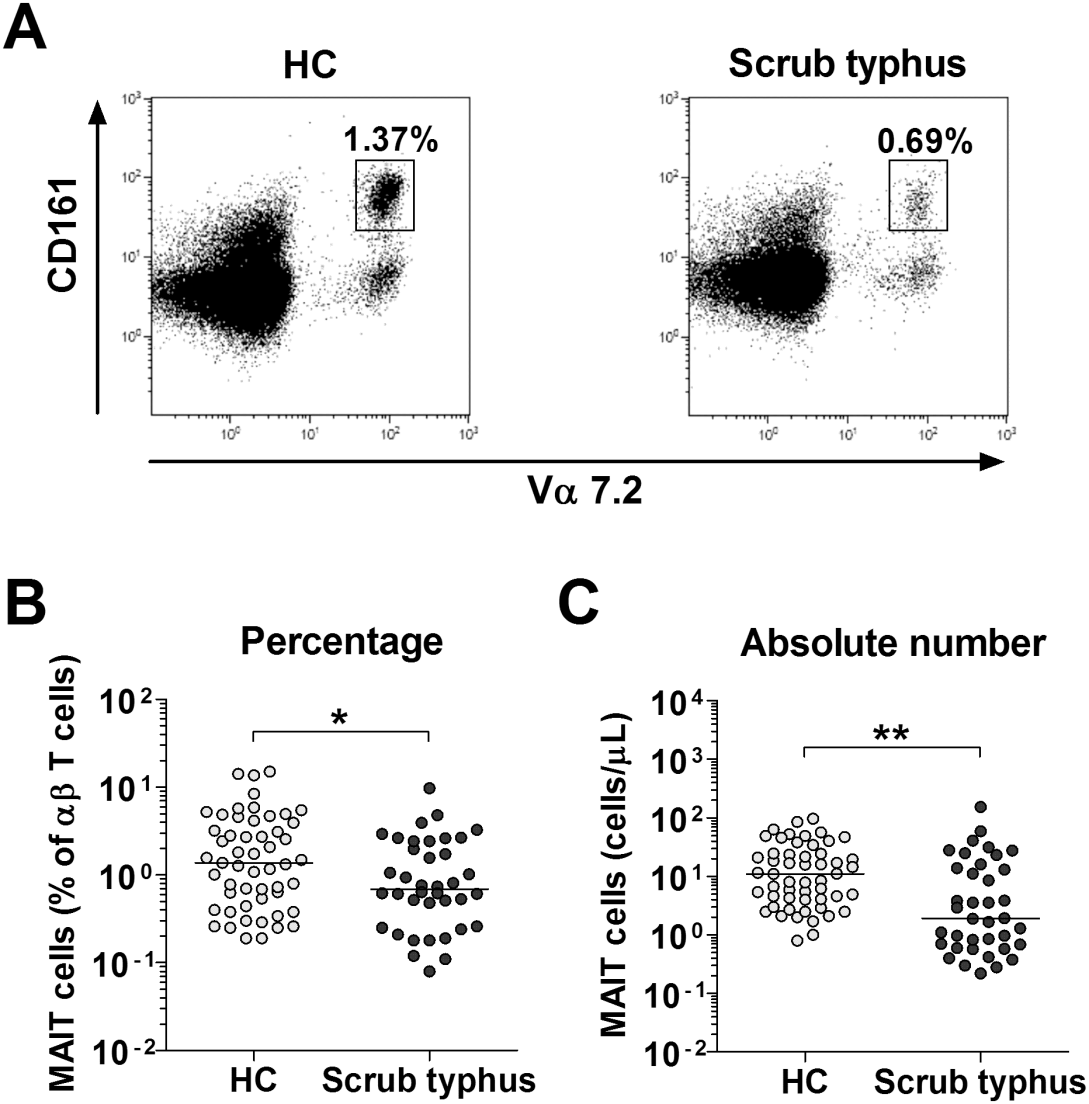
Decreased circulating MAIT cell numbers in the peripheral blood of scrub typhus patients. Freshly isolated PBMCs from 53 HCs and 38 patients with scrub typhus were stained with APC-Alexa Fluor 750-conjugated anti-CD3, FITC-conjugated anti-TCR γδ, APC-conjugated anti-TCR Vα7.2 and PE-Cy5-conjugated anti-CD161 mAbs, and then analyzed by flow cytometry. Percentages of MAIT cells were calculated using a αβ T cell gate. Panel A: Representative MAIT cell percentages as determined by flow cytometry. Panel B: MAIT cell percentages among peripheral blood αβ T cells. Panel C: Absolute MAIT cell numbers (per microliter of blood). Symbols represent individual subjects and horizontal lines are median values. *p < 0.05, **p < 0.001 by the Mann-Whitney U test.

The percentages and absolute numbers of MAIT cells in the peripheral blood samples of 38 patients with scrub typhus and 53 HCs were determined by flow cytometry. MAIT cells were defined as CD3+TCRγδ- cells expressing TCR Vα7.2 and CD161^high^ (Fig 1A). Percentages of MAIT cells were significantly lower in scrub typhus patients than in HCs (median 0.69% versus 1.37% [p < 0.05]) (Fig 1B). Absolute numbers of MAIT cells were calculated by multiplying MAIT cell percentages by the CD3+TCRγδ- T cell percentages and the total lymphocyte numbers (per microliter of peripheral blood). Scrub typhus patients had significantly lower absolute numbers of MAIT cells than HCs (median 1.89 cells/μL versus 11.0 cells/μL [p < 0.001]) (Fig 1C).

### Relationship between circulating MAIT cell levels and clinical parameters in scrub typhus patients

To evaluate the clinical relevance of MAIT cell levels in 38 patients with scrub typhus, we investigated the correlation between MAIT cell percentages in the peripheral blood and clinical parameters by regression analysis (Table 2). The univariate linear regression analysis showed that circulating MAIT cell percentages were significantly correlated with age, alanine aminotransferase level, alkaline phosphatase level, and severity (p = 0.001, p = 0.043, p = 0.009, and p = 0.047, respectively). However, no significant correlation was observed between MAIT cell percentages and leukocyte count, lymphocyte count, hemoglobin level, neutrophil count, platelet count, total bilirubin level, total protein level, albumin level, aspartate aminotransferase level, lactate dehydrogenase level, C-reactive protein level and erythrocyte sedimentation rate (Table 2).

**Table 2:** Regression coefficients of MAIT cell percentages with respect to clinical and laboratory parameters in scrub typhus patients.

Variable	Univariate			Multivariate		
	β	SE	p-value	β	SE	p-value
Age (years)	-0.059	0.017	0.001*	-0.047	0.018	0.014*
Leukocyte count (cells/μL)	0.000	0.000	0.797			
Lymphocyte count (cells/μL)	0.041	0.147	0.783			
Hemoglobin level (g/dL)	0.041	0.147	0.783			
Neutrophil count (cells/μL)	0.000	0.000	0.671			
Platelet count (×10^3^ cells/μL)	0.005	0.005	0.269			
Total bilirubin level (mg/dL)	-0.955	1.169	0.419			
Total protein level (g/dL)	0.597	0.496	0.236			
Albumin level (g/dL)	0.434	0.482	0.375			
AST level (U/L)	0.006	0.004	0.125			
ALT level (U/L)	0.007	0.004	0.043*	0.003	0.003	0.333
Alkaline phosphatase level (U/L)	0.010	0.003	0.009*	0.006	0.003	0.084
LDH level (U/L)	0.000	0.001	0.997			
CRP level (mg/dL)	-0.051	0.042	0.235			
ESR level (mm/hour)	-0.016	0.009	0.075			
Severity	-0.926	0.451	0.047*	-0.109	0.453	0.811

*Abbreviations*: ALT: alanine aminotransferase; AST: aspartate aminotransferase; CRP: C-reactive protein; ESR: erythrocyte sedimentation rate; LDH: lactate dehydrogenase; β: regression coefficients; SE: standard error.

* indicates statistical significance.

### Impaired TNF-α production in MAIT cells from scrub typhus patients

**Fig 2:**
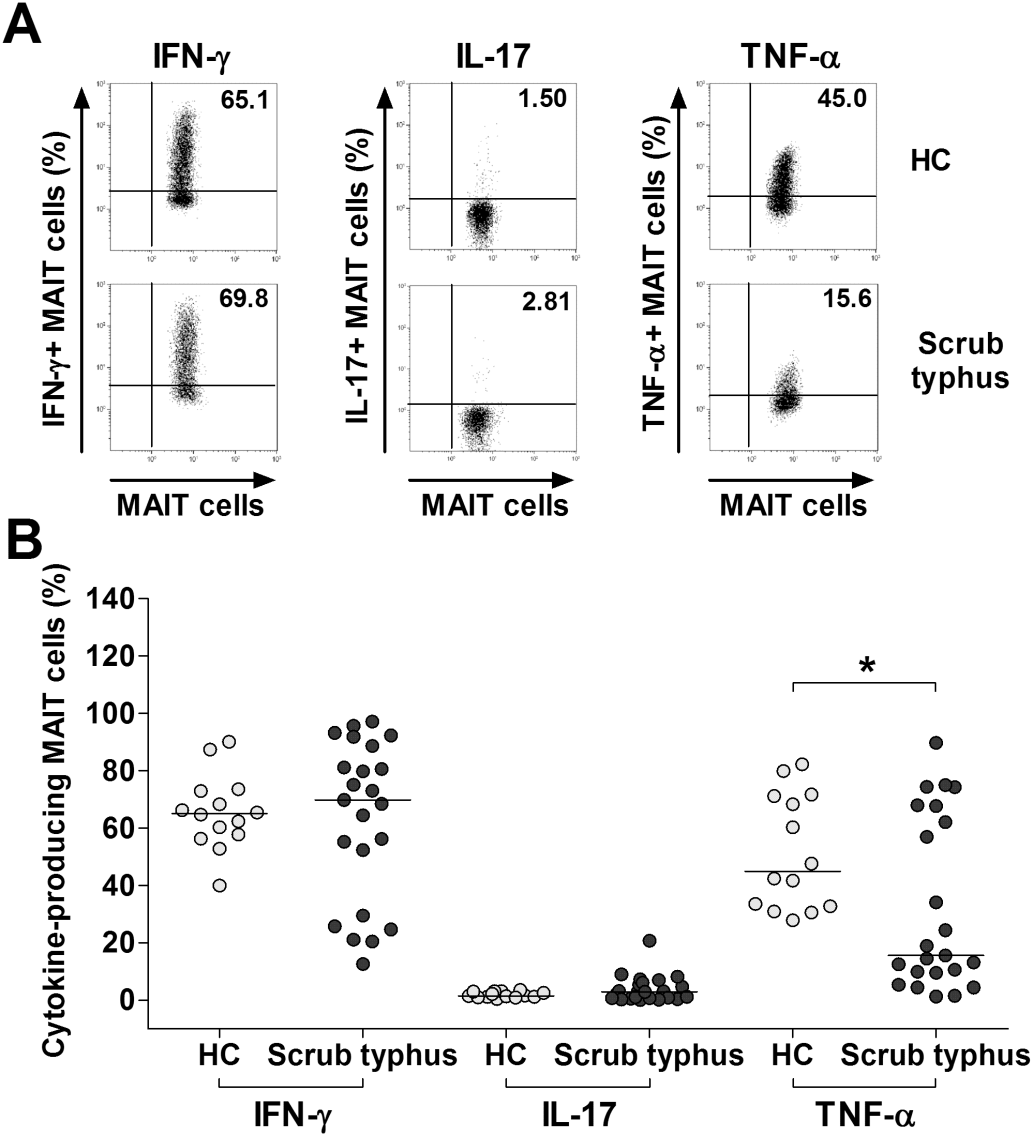
Expression of IFN-γ, IL-17 and TNF-α in MAIT cells from scrub typhus patients. Freshly isolated PBMCs (1 × 10^6^/well) were incubated for 4 hours in the presence of PMA (100 ng/ml) and IM (1 μM). Panel A: Representative IFN-γ, IL-17 and TNF-α expression in the MAIT cell population were determined by intracellular flow cytometry after stimulation with PMA and IM. Data in panel B were obtained from 14 HCs and 23 patients with scrub typhus. Symbols represent individual subjects and horizontal lines are median values. *p < 0.05 by the Mann-Whitney U test.

Hepatocytes and endothelial cells stimulated by proinflammatory cytokines (e.g., IFN-γ and TNF-α) have been known to kill intracellular rickettsiae via inducible nitric oxide synthase expression and nitric oxide-dependent mechanism [22,23]. To investigate the expression of these cytokines in MAIT cells, we incubated PBMCs from 23 patients with scrub typhus and 14 HCs for 4 hours in the presence of PMA and IM; then the expressions of IFN-γ, IL-17A, TNF-α in the MAIT cell populations were examined at the single-cell level by intracellular flow cytometry (Fig 2A). Percentages of TNF-α+ MAIT cells were found to be significantly lower in scrub typhus patients than in HCs (median 15.6% versus 45.0% [p < 0.05]). However, IFN-γ+ or IL-17A+ MAIT cell levels were comparable between scrub typhus patients and HCs (Fig 2B).

### Activation of MAIT cells in scrub typhus patients

**Fig 3:**
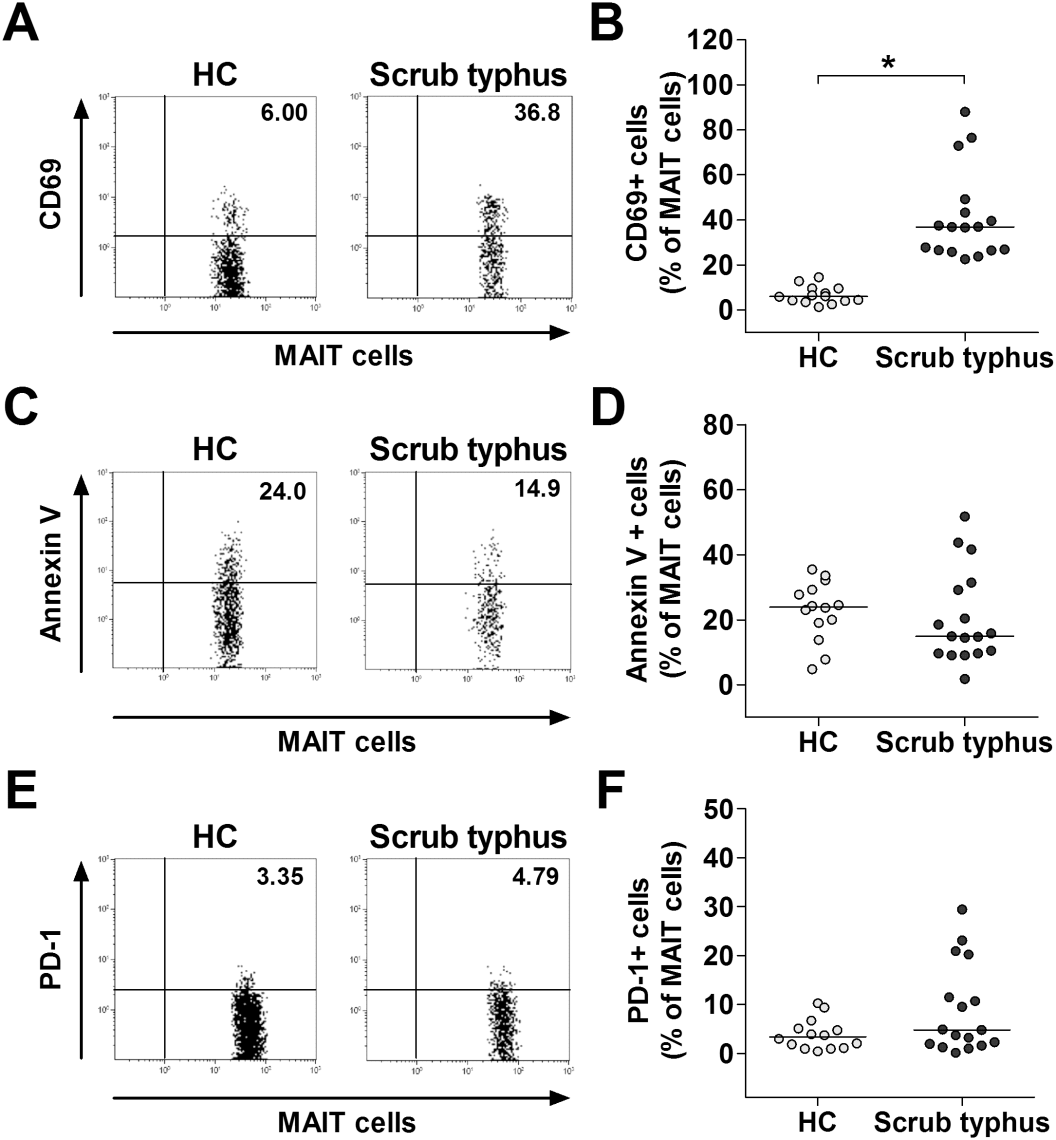
Expression of CD69 and PD-1 and apoptosis of MAIT cells from scrub typhus patients. Freshly isolated PBMCs were stained with FITC-conjugated anti-CD3, FITC-conjugated annexin V, APC-conjugated anti-TCR Vα7.2, PE-conjugated anti-CD3, PE-conjugated anti-CD69, PE-conjugated anti-PD-1 and PE-Cy5-conjugated anti-CD161 monoclonal antibodies, and then analyzed by flow cytometry. Percentages of CD69-expressing cells (panel A), annexin V-positive cells (panel C) and PD-1-expressing cells (panel E) among MAIT cells were determined by flow cytometry. Data in panels B, D and F were obtained from 14 HCs and 17 patients with scrub typhus. Symbols represent individual subjects and horizontal lines are median values. *p < 0.0001 by the Mann-Whitney U test.

The relationship between the expression of immune activation markers and the loss of circulating MAIT cells has been reported in HIV-infected patients [24]. To determine whether MAIT cell deficiency in scrub typhus patients is correlated with activation-induced cell death, CD69+ and annexin V+ cell levels in circulating MAIT cells were determined by flow cytometry. Percentages of CD69+ MAIT cells were found to be significantly higher in scrub typhus patients than in HCs (median 36.8% versus 6.0% [p < 0.0001]) (Fig 3A and 3B). However, annexin V+ cell levels were comparable between scrub typhus patients and HCs (Fig 3C and 3D). PD-1 and its ligands, PD-1 L1 and PD-L2, are known to deliver inhibitory signals that regulate the balance among T cell activation, tolerance and immunopathology [25]. To determine whether impaired TNF-α production by MAIT cells is related to PD-1, we examined the expression levels of PD-1 in the peripheral blood samples of 17 scrub typhus patients and 14 HCs. The expression levels of PD-1 in MAIT cells were similar between scrub typhus patients and HCs (Fig 3E and 3F).

**Fig 4:**
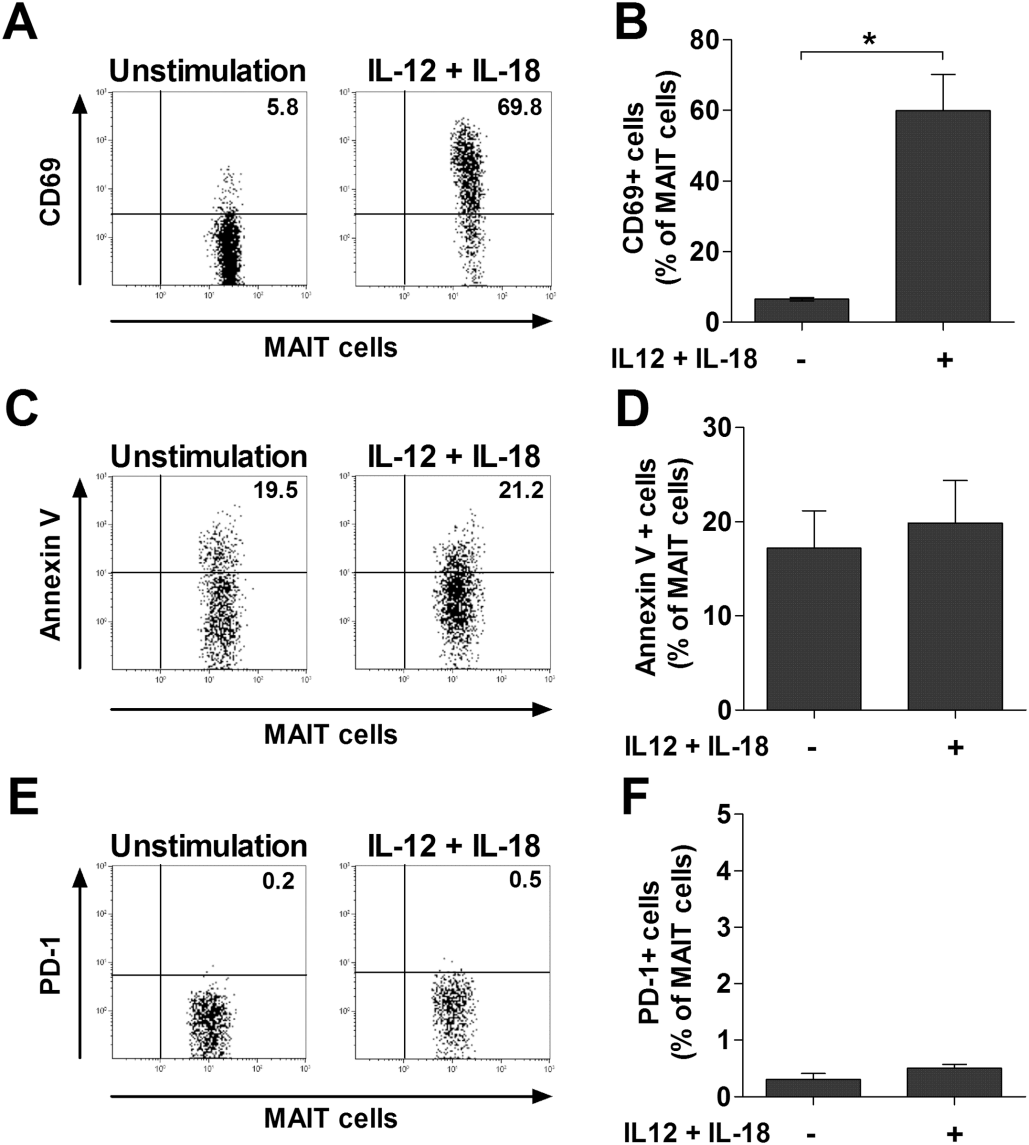
Expression of CD69 and PD-1 and apoptosis of MAIT cells after stimulation with IL-12 and IL-18. PBMCs (1 × 10^6^/well) were incubated for 24 hours in the presence of IL-12 (50 ng/mL) and IL-18 (50 ng/mL), and then stained with FITC-conjugated anti-CD3, FITC-conjugated annexin V, APC-conjugated anti-TCR Vα7.2, PE-conjugated anti-CD3, PE-conjugated anti-CD69, PE-conjugated anti-PD-1 and PE-Cy5-conjugated anti-CD161 monoclonal antibodies. Percentages of CD69-expressing cells (panel A), annexin V-positive cells (panel C), and PD-1-expressing cells (panel E) among MAIT cells were determined by flow cytometry. Data in panels B, D and F were obtained from 6 HCs. Values are expressed as the mean ± SEM. *p < 0.005 by paired t test.

To determine whether MAIT cells can be activated by IL-12 and IL-18, PBMCs from HCs were cultured with IL-12 and IL-18 for 24 hours and then CD69+, annexin V+, and PD-1+ cell levels in MAIT cells were determined by flow cytometry. Percentages of CD69+ MAIT cells were found to be significantly higher in IL-12- and IL-18-treated cultures compared with untreated cultures (mean ± SEM 59.9 ± 10.29% versus 6.5 ± 0.48% [p < 0.005]) (Fig 4A and 4B). However, annexin V+ and PD-1+ MAIT cell levels were comparable between the treated and untreated cultures (Fig 4C and 4F).

### Changes in MAIT cell levels and functions according to disease activity

**Fig 5:**
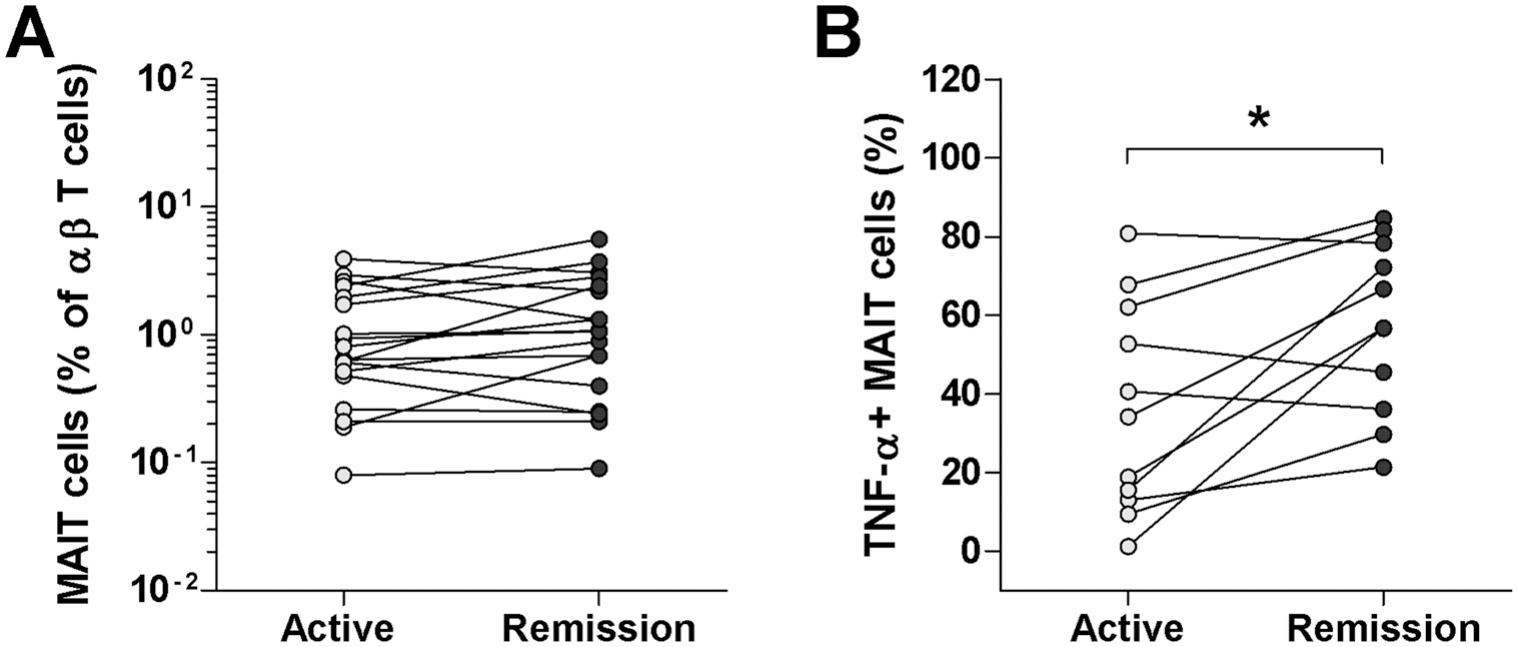
Changes in MAIT cell levels and functions in scrub typhus patients. The percentages of MAIT cells (panel A) in the peripheral blood of 18 scrub typhus patients during active disease and remission were determined by flow cytometry. TNF-α expression (panel B) in the MAIT cell population after stimulation with PMA and IM was determined by intracellular flow cytometry. Data in panel B were obtained from 11 patients with scrub typhus. Symbols represent individual subjects. *p < 0.05 by the Wilcoxon matched-pairs signed rank test.

Based on our observation that circulating MAIT cell levels and TNF-α production are reduced in scrub typhus patients, we investigated the changes in circulating MAIT cell levels and functions in relation to disease activity. Eighteen and eleven patients were available for follow-up examination of MAIT cell levels and functions, respectively. As shown in Fig 5A, no significant changes in circulating MAIT cell levels were found according to disease activity. However, TNF-α+ MAIT cell levels were found to be greater when the disease was in remission than when it was active (median 56.8% versus 34.1% [p < 0.05]) (Fig 5B).

## Discussion

Although the importance of an immune response of MAIT cells in host protection against certain mycobacterial and enterobacterial infections is well established [11,12,15–19], the role of MAIT cells in *O*. *tsutsugamushi* infection remains unclear. To the best of our knowledge, this is the first study to measure the levels and functions of circulating MAIT cells in scrub typhus patients and to examine the clinical relevance of MAIT cell levels. The present study showed that percentages and numbers of circulating MAIT cells were lower in scrub typhus patients than in HCs. We also demonstrated that MAIT cell deficiency reflects disease severity. In particular, *in vitro* experiments showed poor production of TNF-α by MAIT cells during active disease, but TNF-α production was found to be increased during remission. Finally, our study showed that the impaired production of TNF-α by MAIT cells was associated with elevated CD69 expression in scrub typhus patients. These findings possibly contribute to immune system dysregulation in scrub typhus infection and have important implications for the development of cell-based immunotherapy.

In this study, circulating MAIT cell levels were found to be reduced in scrub typhus patients. In contrast, frequencies of other T cell subsets (e.g., CD3 T cells, αβ T cells and γδ T cells) were found to be similar between the patients and HCs (S1 Fig), suggesting that the decline in cell levels is specific to MAIT cells. MAIT cell deficiency in peripheral blood has also been reported in several other infectious diseases, including *Vibrio cholerae* O1 infection, *Pseudomonas aeruginosa* infection in cystic fibrosis, and severe bacterial sepsis, in particular non-streptococcal infection [26–28]. Our previous study revealed that MAIT cells were numerically and functionally deficient in patients with pulmonary tuberculosis and nontuberculous mycobacteria lung disease [19]. In the present study, circulating MAIT cells were more reduced in patients with organ dysfunction than in patients without organ dysufunction (S2 Fig). It is well known that scrub typhus occurs when the host is bitten by an *O*. *tsutsugamushi*-infected trombiculid mite and it can spread to other organs via the bloodstream (e.g., kidney, liver, lung and brain) [2]. Thus, the decline in circulating MAIT cell numbers might be due to migration of MAIT cells from the peripheral circulation to infected tissues and organs, for defending against *O*. *tsutsugamushi* infection.

The present study revealed that MAIT cell percentages in the peripheral blood were significantly correlated with age, alanine aminotransferase level, alkaline phosphatase level, and disease severity. Consistent with our data, a recent study demonstrated that MAIT cell deficiency was associated with increased disease severity in cystic fibrosis patients with *P*. *aeruginosa* infection [27]. Furthermore, these findings are supported by our previous studies which showed that MAIT cell deficiency is related to the disease severity or extent in patients with chronic obstructive pulmonary disease and mycobacterial infection [19,29]. Collectively, circulating MAIT cell levels may reflect the inflammatory activity or severity of infectious diseases. However, after multivariate analysis, only age was found to be a significant independent predictors of MAIT cell deficiency in scrub typhus patients (Table 2). Similarly, circulating MAIT cell levels were found to be considerably affected by age, irrespective of the healthy or disease state [21,30,31]. Therefore, age-dependent changes in MAIT cell levels could be explained partially by the fact that old age is one of the risk factors affecting the complication and mortality in scrub typhus or other infectious diseases [32,33]. Interestingly, we also found that liver function abnormalities, especially in alanine aminotransferase and alkaline phosphatase, were positively correlated with the MAIT cell levels. Liver is known to be a site of accumulation of MAIT cells and abnormal liver function is frequently observed in scrub typhus patients [34,35]. Therefore, further studies are needed to investigate the integrated role of MAIT cells in hepatic injury.

The fundamental role of TNF-α, one of the cytokines secreted by the Th1 subset, in control of intracellular growth of *O*. *tsutsugamushi* is well established [36,37]. In the present study, the production of TNF-α by MAIT cells was found to be diminished in scrub typhus patients, whereas no significant differences were found between scrub typhus patients and HCs in the production of IFN-γ or IL-17. These findings suggest that the dysregulation of TNF-α production may have a certain role in the pathogenesis of scrub typhus. However, it is not clear why TNF-α secretion by MAIT cells was reduced in response to PMA/IM stimulation. One possible explanation is that impaired TNF-α production by MAIT cells may be due to anergy or exhaustion of these cells during infection. These findings have also been previously observed in other infectious diseases that showed upregulation of coinhibitory receptors [19,24,38]. Another possibility is that the impairment of TNF-α production represents the susceptibility of individuals to *O*. *tsutsugamushi* infection. Interestingly, a previous study revealed that *O*. *tsutsugamushi* inhibited TNF-α production by inducing IL-10 secretion in murine macrophages [39]. Third possibility is that high levels of proinflammatory cytokines can induce such apparent reduced responsiveness of MAIT cells. This hypothesis is supported by our supplementary data showing that plasma levels of proinflammatory cytokines, such as IFN-γ and TNF-α, were found to be significantly higher in scrub typhus patients than in HCs (S3 Fig), which was consistent with the results of previous study [40]. Moreover, these similar findings have also been observed in our previous study treating another invariant T cells (e.g., natural killer T cells) with IL-1β, IL-6, IL-8, IL-18, IFN-γ and TNF-α [41]. Further studies are needed to prove this hypothesis in MAIT cells. Altogether, these results indicate that impaired TNF-α production by MAIT cells is due to a negative-feedback mechanism that allows the pathogen to survive in the hostile environment.

MAIT cells have been known to be stimulated either in an MR1-dependent manner or in an MR1-independent manner [42]. Our study showed that MAIT cells were activated in scrub typhus patients, indicated by CD69 up-regulation. According to the Kyoto Encyclopedia of Genes and Genomes (KEGG) at http://www.kegg.jp/kegg/pathway.html, none of *O*. *tsutsugamushi* Boryong and Ikeda strains harbor the riboflavin biosynthesis pathway. Until proven otherwise, this means that this bacterial strain (especially, Boryong, most common strain in South Korea) does not provide the MAIT cell MR1 ligand, suggesting that MAIT cells can be activated in MR1-independent manner. It has been previously reported that MAIT cells highly express IL-18R and IL-12R [43,44]. In accordance with the results of previous studies [40,43], the present study demonstrated that MAIT cells were activated after stimulation by IL-12 and IL-18. Furthermore, a previous study showed that plasma IL-12p40 and IL-18 levels were elevated in scrub typhus patients [40]. Collectively, these findings suggest that MAIT cells can be activated by IL-12 and IL-18, in an MR1-independent manner, in scrub typhus.

CD69 is known to be early activation marker, whereas PD-1 is considered as relative late activation marker. Following TCR activation, CD69 is upregulated within ~4 hours [45], whereas PD-1 is upregulated within 24–72 hours [46]. PD-1 expression in circulating MAIT cells is also known to be upregulated in chronic infectious diseases, such as chronic human immunodeficiency virus, tuberculosis, and chronic hepatitis C virus infections [19,47,48]. In addition, recent studies reported that the PD-1 plays a complex role during acute infection [46]. However, little is known about the role of PD-1 in acute bacterial infection, particularly in scrub typhus. Investigation of CD69 and PD-1 expression on MAIT cells can provide information on the time course of disease activation during *O*. *tsutsugamushi* infection. In the current study, circulating MAIT cells were found to be markedly activated, as indicated by the upregulation of CD69 expression, while marginal expression of PD-1 was observed in a subset of acute scrub typhus patients, indicating that MAIT cells may play an important role in early phase during scrub typhus infection. In line with our data, Doe et al recently reported that CD4+ T cells from mice infected with *Plasmodium* parasites expressed PD-1 as early as 6 days after infection, while *Listeria monocytogenes* induced marginal expression of PD-1 [49]. These results imply that the mode and function of PD-1 expression might differ between acute infection and chronic infection, i.e., PD-1 expression may occur in the late stage of infection or may depend on the microbes. In addition, our data showed that PD-1+ MAIT cell levels were inversely correlated with the corresponding TNF-α production by MAIT cells from the patients (S4 Fig), suggesting that functional impairment of MAIT cells may partially be due to the negative regulation including PD-1. Another interesting finding from our study is that the levels of apoptotic T cells, defined as annexin V+ cells, were comparable between scrub typhus patients and HCs. These results indicate that reduced MAIT cells in peripheral blood might be due to their migration to the infected tissue or organs, rather than MAIT cell death.

In the present study, impairment in TNF-α production of MAIT cells in the acute phase of scrub typhus infection was found to be restored during the remission phase, while numerical deficiency of MAIT cells did not change over time. Consistent with our data, a previous study demonstrated that the function of MAIT cells was restored partially after effective antiretroviral therapy, although levels of MAIT cells in peripheral blood were not restored [24]. A similar finding about MAIT cell levels in peripheral blood has also been reported in pediatric patients with *Vibrio cholerae* O1 infection [26]. Circulating MAIT cell levels were significantly reduced at day 7 and this deficiency persisted up to 90 days after onset of cholera. In a recent study, circulating MAIT cell numbers started to increase as early as 4 days after severe sepsis, but persistent depletion of MAIT cells was found in some patients, depending on their clinical condition [28]. Thus, further long-term follow-up studies are needed to determine when circulating MAIT cell levels are restored after scrub typhus infection.

In summary, the present study demonstrates that circulating MAIT cells are activated, numerically deficient, and functionally impaired in TNF-α production in patients with scrub typhus. In addition, MAIT cell deficiency reflects disease severity. These findings provide important information for predicting the prognosis of scrub typhus infection.

## Supporting Information

S1 Fig Frequencies and function of T cell subsets in scrub typhus patients. Panel A: Percentages of T cell subsets in scrub typhus patients. Freshly isolated PBMCs from 53 HCs and 38 patients with scrub typhus were stained with APC-Alexa Fluor 750-conjugated anti-CD3, FITC-conjugated anti-TCR γδ, and then analyzed by flow cytometry. Percentages of CD3 T cells, αβ T cells and γδ T cells were calculated using lymphocytes gate. Panel B: Cytokine production of T cells in scrub typhus patients. PBMCs (1 × 10^6^/well) from 14 HCs and 23 patients with scrub typhus were incubated for 4 hours in the presence of PMA (100 ng/ml) and IM (1 μM). Production of IFN-γ, IL-17 and TNF-α by CD3 T cells was measured by intracellular flow cytometry. Symbols represent individual subjects and horizontal lines are median values. NS = not significant by the Mann-Whitney U test.
(TIF)

S2 Fig Reduced circulating MAIT cell percentages in patients with organ dysfunction. Freshly isolated PBMCs from 38 patients with scrub typhus were stained with APC-Alexa Fluor 750-conjugated anti-CD3, FITC-conjugated anti-TCR γδ, APC-conjugated anti-TCR Vα7.2 and PE-Cy5-conjugated anti-CD161 mAbs, and then analyzed by flow cytometry. Based on the number of dysfunctional organs, the severity of scrub typhus can be subclassified into mild (no organ dysfunction), moderate (one organ dysfunction), and severe (dysfunction of two or more organs) diseases. Percentages of MAIT cells were calculated using a αβ T cell gate. Symbols represent individual subjects and horizontal lines are median values. *p < 0.05, by the Mann-Whitney U test.
(TIF)

S3 Fig Plasma levels of IFN-γ, IL-17 and TNF-α in patients with scrub typhus. Plasma samples of the patients were collected before specific treatment on admission. Plasma levels of IFN-**γ** (panel A), IL-17 (panel B) and TNF-α (panel C) were determined by Luminex. Data were obtained from 8 HCs and 22 patients with scrub typhus. Symbols represent individual subjects and horizontal lines are median values. *p < 0.05, **p < 0.01 by the Mann-Whitney U test.
(TIF)

S4 Fig Relationship between TNF- production and PD-1 expression in MAIT cells from scrub typhus patients. The correlations between TNF-+ cell levels and PD-1+ cell levels in MAIT cells were examined using Spearman's correlation analysis. TNF-+ MAIT cell percentages were negatively correlated with PD-1+ MAIT cell percentages (γ = -0.6484, p < 0.05). Symbols represent individual subjects.
(TIF)

S5 Fig Frequencies and function of MAIT cells in patients with influenza viral infection. Panel A: Percentages of circulating MAIT cells in patients with influenza viral infection. Freshly isolated PBMCs from 10 HCs and 10 patients with influenza viral infection were stained with APC-Alexa Fluor 750-conjugated anti-CD3, FITC-conjugated anti-TCR γδ, APC-conjugated anti-TCR Vα7.2 and PE-Cy5-conjugated anti-CD161 mAbs and then analyzed by flow cytometry. Percentages of MAIT cells were calculated using a αβ T cell gate. Panel B: Cytokine production of T cells in patients with influenza viral infection. PBMCs (1 × 10^6^/well) from 10 HCs and 10 patients with influenza viral infection were incubated for 4 hours in the presence of PMA (100 ng/ml) and IM (1 μM). Production of IFN-γ, IL-17 and TNF-α by MAIT cells was measured by intracellular flow cytometry. Symbols represent individual subjects and horizontal lines are median values. NS = not significant by the Mann-Whitney U test.
(TIF)

S1 Checklist STROBE Checklist. (DOC)
