# Supplementary figures and images for: Activation, Impaired Tumor Necrosis Factor-α Production, and Deficiency of Circulating Mucosal-Associated Invariant T Cells in Patients with Scrub Typhus
Source: PLoS Negl Trop Dis. 2016 Jul 27;10(7):e0004832. doi: 10.1371/journal.pntd.0004832 (PMC4963088; doi:10.1371/journal.pntd.0004832)

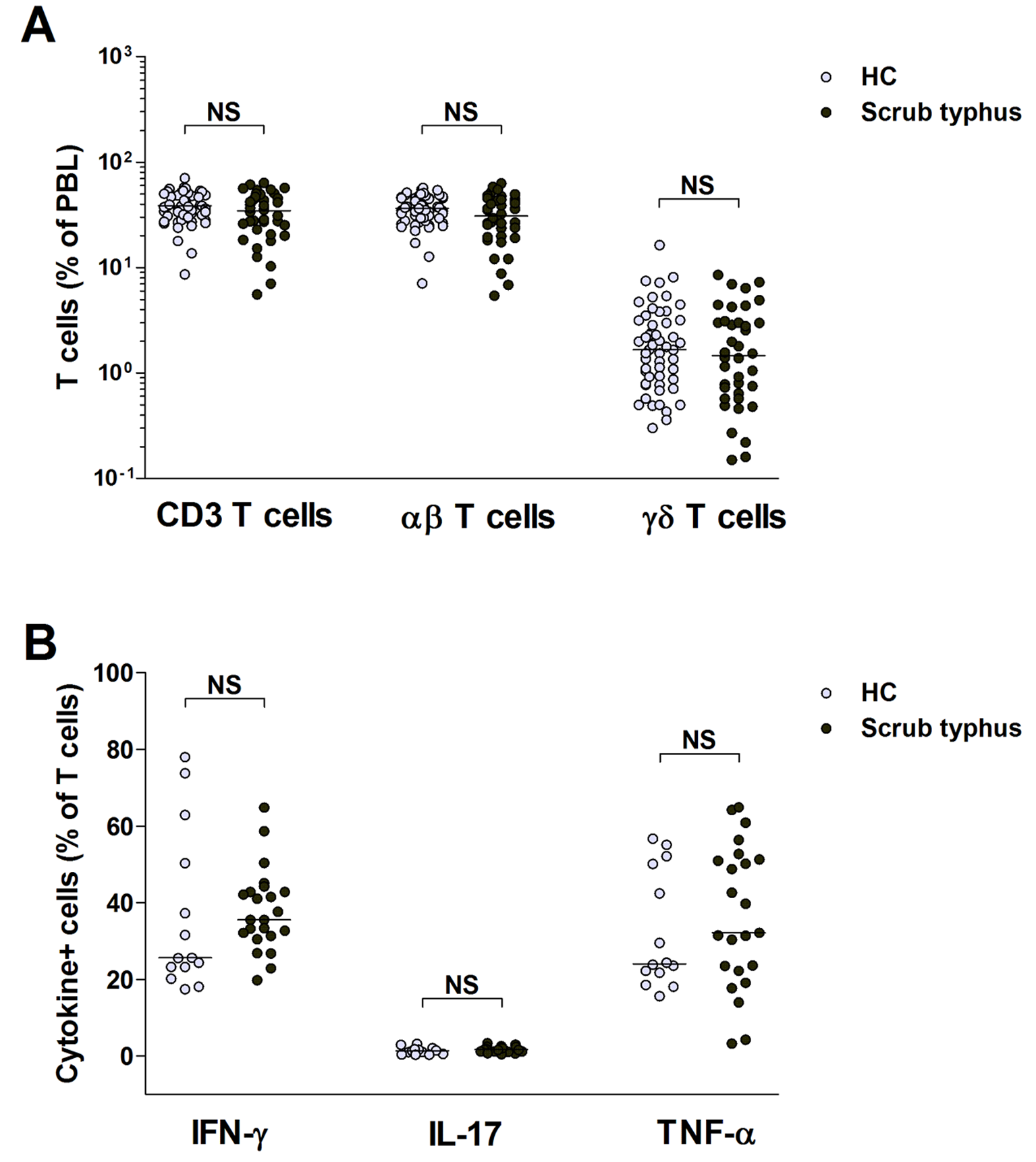

Supplement: S1 Fig — Panel A: Percentages of T cell subsets in scrub typhus patients. Freshly isolated PBMCs from 53 HCs and 38 patients with scrub typhus were stained with APC-Alexa Fluor 750-conjugated anti-CD3, FITC-conjugated anti-TCR γδ, and then analyzed by flow cytometry. Percentages of CD3 T cells, αβ T cells and γδ T cells were calculated using lymphocytes gate. Panel B: Cytokine production of T cells in scrub typhus patients. PBMCs (1 × 10^6^/well) from 14 HCs and 23 patients with scrub typhus were incubated for 4 hours in the presence of PMA (100 ng/ml) and IM (1 μM). Production of IFN-γ, IL-17 and TNF-α by CD3 T cells was measured by intracellular flow cytometry. Symbols represent individual subjects and horizontal lines are median values. NS = not significant by the Mann-Whitney U test. (TIF) [file pntd.0004832.s001.tif]

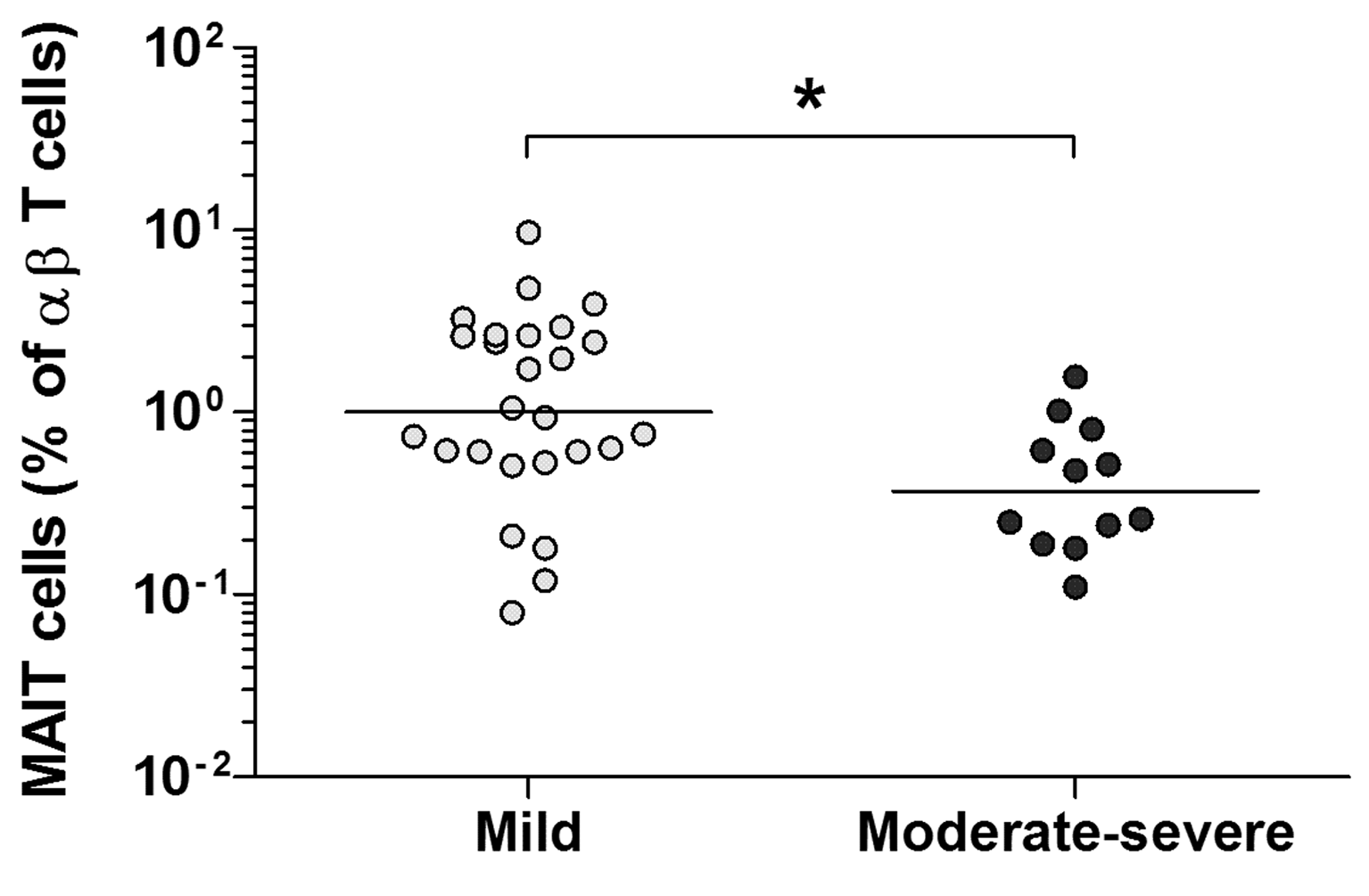

Supplement: S2 Fig — Freshly isolated PBMCs from 38 patients with scrub typhus were stained with APC-Alexa Fluor 750-conjugated anti-CD3, FITC-conjugated anti-TCR γδ, APC-conjugated anti-TCR Vα7.2 and PE-Cy5-conjugated anti-CD161 mAbs, and then analyzed by flow cytometry. Based on the number of dysfunctional organs, the severity of scrub typhus can be subclassified into mild (no organ dysfunction), moderate (one organ dysfunction), and severe (dysfunction of two or more organs) diseases. Percentages of MAIT cells were calculated using a αβ T cell gate. Symbols represent individual subjects and horizontal lines are median values. *p < 0.05, by the Mann-Whitney U test. (TIF) [file pntd.0004832.s002.tif]

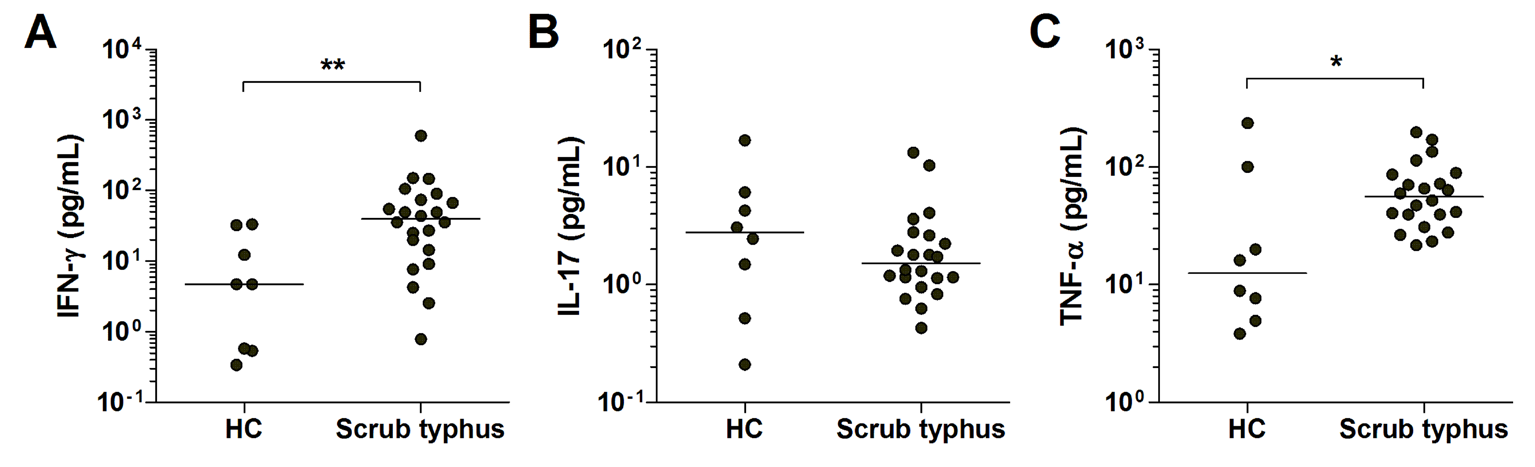

Supplement: S3 Fig — Plasma samples of the patients were collected before specific treatment on admission. Plasma levels of IFN-γ (panel A), IL-17 (panel B) and TNF-α (panel C) were determined by Luminex. Data were obtained from 8 HCs and 22 patients with scrub typhus. Symbols represent individual subjects and horizontal lines are median values. *p < 0.05, **p < 0.01 by the Mann-Whitney U test. (TIF) [file pntd.0004832.s003.tif]

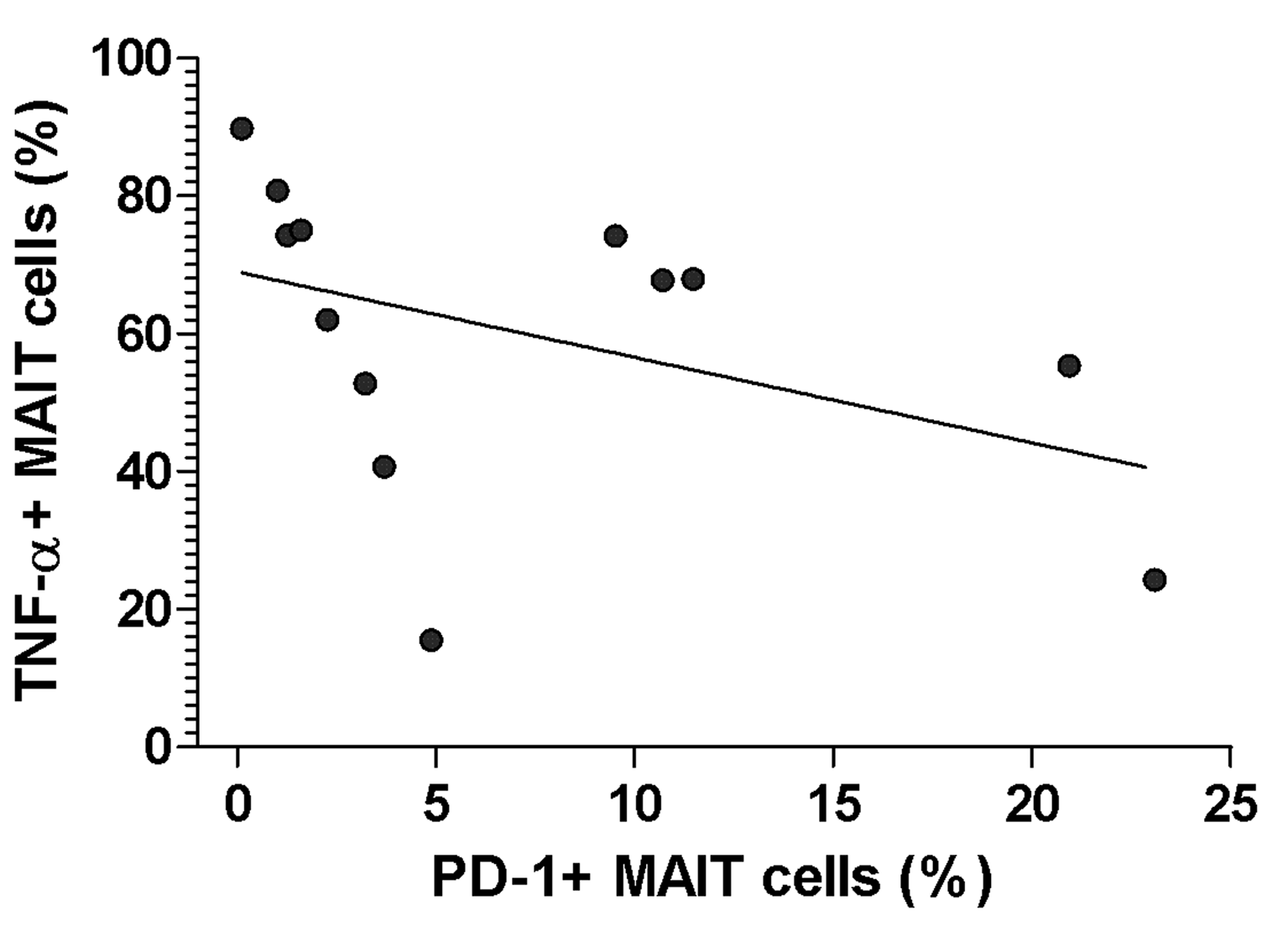

Supplement: S4 Fig — The correlations between TNF-+ cell levels and PD-1+ cell levels in MAIT cells were examined using Spearman's correlation analysis. TNF-+ MAIT cell percentages were negatively correlated with PD-1+ MAIT cell percentages (γ = -0.6484, p < 0.05). Symbols represent individual subjects. (TIF) [file pntd.0004832.s004.tif]

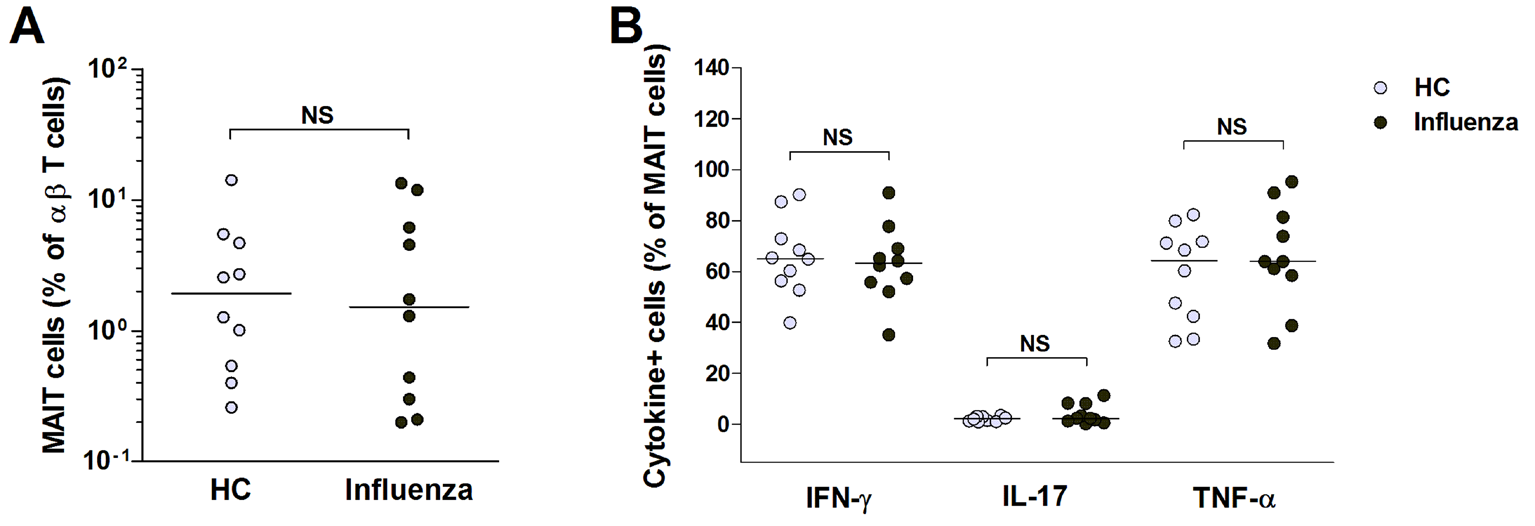

Supplement: S5 Fig — Panel A: Percentages of circulating MAIT cells in patients with influenza viral infection. Freshly isolated PBMCs from 10 HCs and 10 patients with influenza viral infection were stained with APC-Alexa Fluor 750-conjugated anti-CD3, FITC-conjugated anti-TCR γδ, APC-conjugated anti-TCR Vα7.2 and PE-Cy5-conjugated anti-CD161 mAbs and then analyzed by flow cytometry. Percentages of MAIT cells were calculated using a αβ T cell gate. Panel B: Cytokine production of T cells in patients with influenza viral infection. PBMCs (1 × 10^6^/well) from 10 HCs and 10 patients with influenza viral infection were incubated for 4 hours in the presence of PMA (100 ng/ml) and IM (1 μM). Production of IFN-γ, IL-17 and TNF-α by MAIT cells was measured by intracellular flow cytometry. Symbols represent individual subjects and horizontal lines are median values. NS = not significant by the Mann-Whitney U test. (TIF) [file pntd.0004832.s005.tif]
